# Value Cocreation in Health Care: Systematic Review

**DOI:** 10.2196/33061

**Published:** 2022-03-25

**Authors:** Yuxin Peng, Tailai Wu, Zhuo Chen, Zhaohua Deng

**Affiliations:** 1 School of Medicine and Health Management Tongji Medical College Huazhong University of Science and Technology Wuhan China; 2 Department of Health Policy and Management University of Georgia College of Public Health Athens, GA United States; 3 School of Economics, Faculty of Humanities and Social Sciences University of Nottingham Ningbo China Ningbo China; 4 School of Management Huazhong University of Science and Technology Wuhan China

**Keywords:** value cocreation, health care, patient value, health care professional value, systematic review

## Abstract

**Background:**

Value cocreation in health care (VCCH), mainly based on service-dominant logic, emphasizes that participants, including both patients and physicians, can effectively enroll in the health care value creation process. Effective VCCH is of great significance for realizing value-based health care and improving doctor-patient relationships. Therefore, a comprehensive understanding of VCCH is critical. However, the current literature on VCCH is fragmented and not well studied.

**Objective:**

The goal of the research is to investigate the antecedents, consequences, and dimensions of VCCH by systematically searching, selecting, summarizing, and evaluating relevant literature.

**Methods:**

English-language articles on VCCH in the Web of Science, PubMed, and Scopus databases published from January 2008 to December 2019 were identified. The articles were screened using the Preferred Reporting Items for Systematic Reviews and Meta-Analyses protocol, and the quality of studies included were appraised using the Mixed Methods Appraisal Tool.

**Results:**

Out of the 181 publications initially identified through the bibliographic searches, 28 publications met the inclusion criteria. This review summarizes antecedents, consequences, and dimensions of VCCH, as well as possible associations among them. An integrative framework is also proposed for mapping the literature of VCCH grounded on social cognitive theory to reveal the whole process of VCCH.

**Conclusions:**

The findings of this systematic review provide implications for continued development of VCCH and contribute to inspire more research in the future.

## Introduction

Value cocreation, which has received much academic attention in the 21st century, describes cooperation among multiple stakeholders [1,2]. As the main theoretical foundation of value cocreation, service-dominant logic (SDL) suggests that the service provider should not be the only value creator, and service receivers could also cocreate the service value with service provider [3-5]. Since service could be a process of interaction between an organization and its customers, value of service based on resources used and integrated in the service process is created by the participants together [3]. Therefore, SDL focuses on value from use rather than value from exchange, which shifts the research focus from goods to services. In recent years, value cocreation is applied in the health care area to understand the patient value creation process.

In health care, value cocreation should be emphasized. Value cocreation in health care (VCCH) refers to the integration of resources through activities and interactions with collaborators to realize the benefit of patients in the health care service delivery network [6]. Therefore, patients and health care providers integrate knowledge, skills, equipment, medicine, facilities, and financial resources to obtain their mutual benefits [7,8]. This definition emphasizes the active participation of patients, who are no longer passive recipients of services but active cocreators. VCCH involves a range of activities around patients or collaboration with service network members, including family members, friends, other patients, health care professionals, and external communities [6]. When patients cocreate health care value with physicians, they participate in the whole processes of treatment. They could offer opinions to improve their compliance [9], eventually improving their health status and service experience [10]. Meanwhile, cocreation between patients and health professionals could also reduce medical costs [11], improve the efficiency of the use of existing medical resources [12,13], and integrate medical resources from different sources to create values with health care professionals [2].

Much of the literature on value cocreation is found in the business field, focusing on the firm and consumer values, yet patient value has attracted attention in the health care field with the changing physician-patient relationship. Patients are increasingly taking an active role in the health care decision-making process [14] and creating value with other participants in the health care delivery process [15]. The relevant notion of value-based health care emphasizes the importance of listening to the patient’s voice and advocates providing high value to patients by taking into account their outcomes, needs, and costs when treating their illnesses [16]. However, value-based health care research touches less on the value creation process. Therefore, it is essential to discuss health care value from a value cocreation perspective.

However, for VCCH, the factors are not explored systematically, underlying mechanisms of its factors are vague, and consequences are not fully investigated. To enrich the research related to VCCH and seek solutions to these known issues, we conducted a systematic review to search, select, and evaluate relevant literature, summarize the theories and methods used in existing studies, and explore the antecedents and consequences of VCCH and the dimensions of value cocreation in previous studies to lay a foundation for future studies.

In this study, the existing relevant literature on VCCH was screened following the Preferred Reporting Items for Systematic Reviews and Meta-Analyses (PRISMA) framework (Multimedia Appendix 1). First, we discuss the development of value cocreation concepts. Second, we describe the process of literature inclusion and selection. Third, we described the available studies, year of publication of the VCCH papers, and the methodological and theoretical underpinnings of the VCCH research. We then summarize the antecedents and consequences of VCCH and value cocreation dimensions from different settings and actors and propose an integrative framework for understanding value cocreation behavior. Finally, we discuss implications, opportunities for future research, and limitations.

## Methods

For this study, we used Web of Science as the primary database and PubMed and Scopus as supplementary databases for literature retrieval conducted on May 25, 2020. Keywords, relevant synonyms, and associated truncations used in the search revolved around two concepts—health care area and value cocreation. The search strategy using topic and Boolean/phrase search modes was used to retrieve papers published from 2008 to 2019 and comprised 3 search strings:

1. patient* OR health OR medical OR “health care” OR “healthcare” OR “online health community*” OR eHealth OR E-health OR mHealth OR m-health OR “mobile health”
2. “value co-creat*” OR “value cocreat*” OR “value-co-creat*” OR “co-creat* of value” OR “co-creat* value”
3. 1 and 2

PRISMA framework was used to record the review process. Initial search results yielded 181 papers across the databases. After duplicates and other types of papers (eg, meeting papers, book chapters) were removed, 134 relevant works were screened. Title and abstract screenings were undertaken independently by two trained research assistants, with disagreements about inclusion resolved through discussion with the research coordinator until an agreement was reached. Papers with titles and abstracts not meeting the selection criteria were removed. Inclusion criteria were empirical studies on value cocreation activities between patients and other participants, including doctors, nurses, and other patients, and studies on the value creation process. Exclusion criteria were non-English language papers; meeting papers, books, editorials, news, reports, and patents; unrelated or incomplete papers; and studies not occurring around patients or in the health care domain. A manual search was conducted of each paper’s reference list to identify relevant papers not recognized in database searches. Finally, 43 papers remained for full-text review.

**Figure 1:**
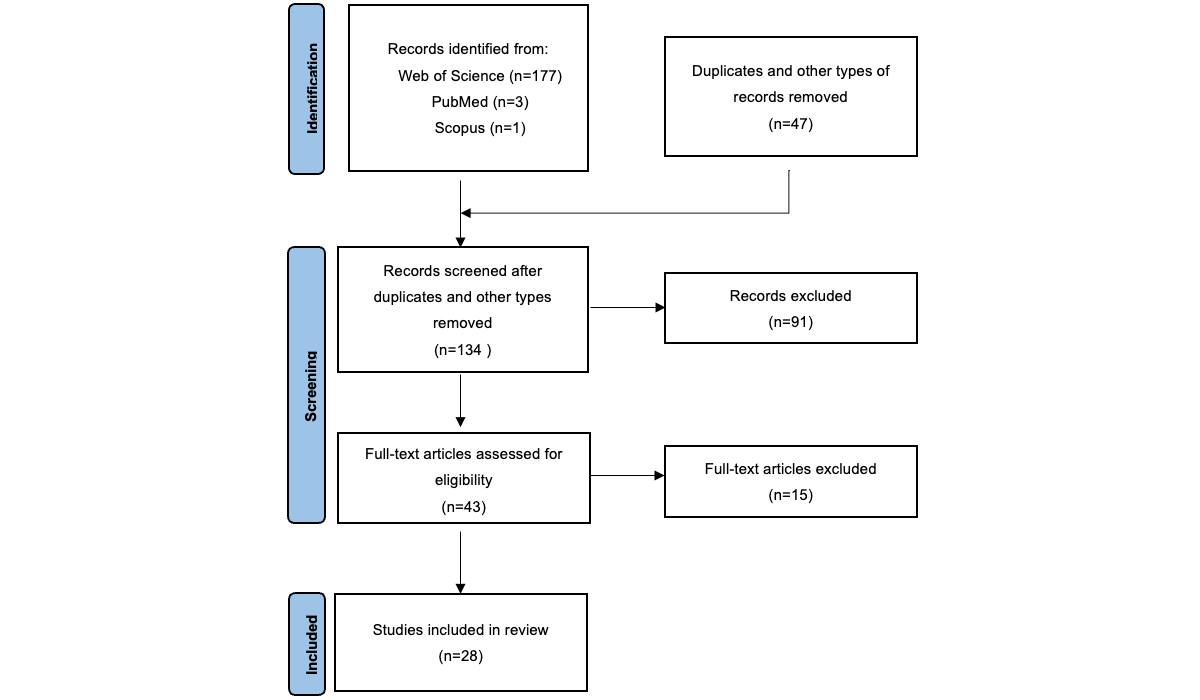
Preferred Reporting Items for Systematic Reviews and Meta-Analyses flow diagram of the screening process.

The selection criteria were applied to the 43 papers reviewed in full in this paper, and the final number of papers included in this review was 28. A flowchart summarizing the search, screening, and study selection process is shown in Figure 1. We used the Mixed Methods Appraisal Tool (MMAT) developed by Pace et al [17] for appraisal of the quality of qualitative, quantitative, and mixed methods research studies included. The results showed that the quality satisfies the requirements of a systematic review; details can be found in Multimedia Appendix 2.

## Results

According to our systematic literature review, we received results in 5 main areas: (1) publication years and journals of existing studies, (2) methods used in existing studies, (3) theories applied in existing studies, (4) dimensions of VCCH in existing studies, and (5) antecedents and consequences of VCCH in existing studies.

### Publication Years and Journals

**Figure 2:**
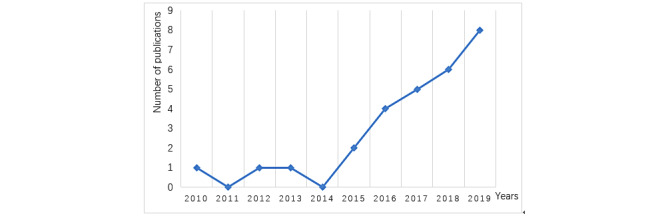
Publication timeline of literature.

As shown in Figure 2, since 2010, there has been a clear upward trend in the research on VCCH. This trend indicates that the application of value cocreation in the medical field is gradually gaining academic attention and is an emerging research area. Following this trend, we expect that more and more studies about VCCH will appear in the coming years.

Toward the publication journals, we found the 28 papers appeared in 17 journals (Multimedia Appendix 3), with 6 journals publishing more than 2 papers on VCCH, indicating that these journals are more interested in this topic. The most publications are in the Journal of Service Management, with 4 studies related to VCCH. The Journal of Service Theory and Practice, Service Business, and Sustainability has published 3 related studies. As can be seen from this distribution, most current research on VCCH was published in journals in the service management field. Meanwhile, some journals in the public health field have published papers on VCCH, including the International Journal of Environmental Research and Public Health and the International Journal of Pharmaceutical and Healthcare Marketing.

### Research Methods

**Figure 3:**
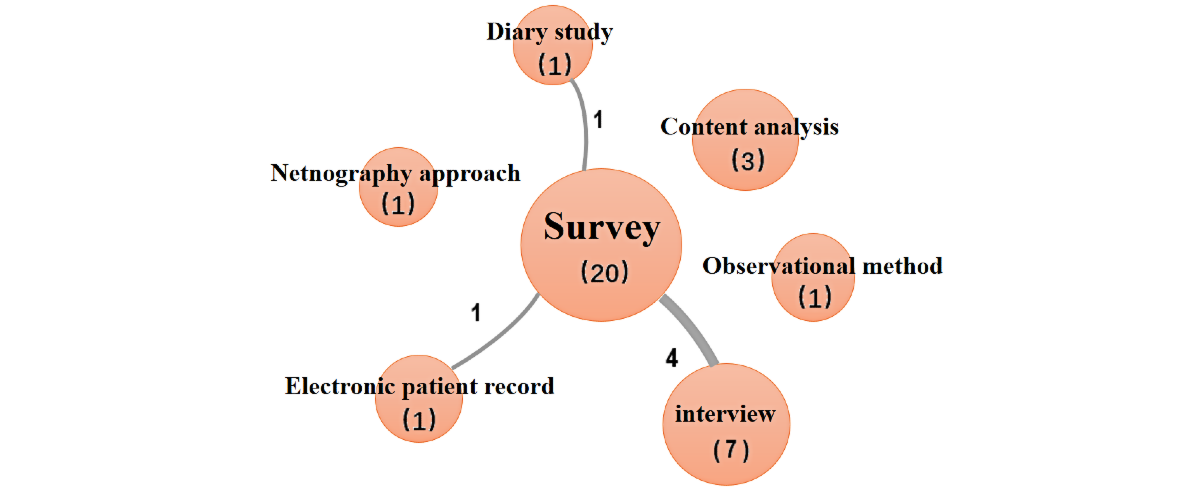
Research methods in the literature.

As shown in Figure 3, survey is the research method most used (20/28, 71%). Several studies used survey along with other methods, including interview (depth interview and semistructured depth interview), diary study, and electronic patient record. We found that survey method combined more with interview method (4 times) than any other methods. This suggests that the interview method allows for a more detailed understanding of participant attitudes and motivations, which facilitates accurate quantitative study. In addition, content analysis, observation, and netnography, an adaptation of ethnography for the contingencies of online community and culture, were used separately in some studies.

### Theories

In the studies included, theories are used in two issues: driving factors of value cocreation behavior/activities and effects of value cocreation behavior/activities (Multimedia Appendix 4).

On driving factors of value cocreation behavior/activities, patient-related, platform-related, and hospital-related factors are proposed to impact value cocreation. For the driving of patient-related factors, several theories including SDL, consumer culture theory, broaden-and-build theory of positive emotions, social identity theory, self-determination theory, social cognitive theory, practice theory, and construal level theory are applied. For the driving of platform factors, theories like organizational support theory and self-awareness theory are leveraged. For the driving of hospital-related factors, customer training and education perspective are used to understand the effects.

Regarding the effects of value cocreation behavior/activities, self-regulation theory is used to explain the effects of value cocreation on several health outcomes such as well-being, health condition, and health care values.

### Dimensions of Value Cocreation in Health Care

In settings where VCCH occurs, different participants are involved in value cocreation with different value creation behaviors. To better understand the dimensions of VCCH, we categorized the literature of VCCH based on the places where VCCH happens and whether VCCH involves the internet. On the places where VCCH happens, since hospitals are the significant medical institutions, we classify VCCH into 2 types based on a typology of VCCH activities: inside-hospital and outside-hospital [18]. With the application of health internet technology, value creators including patients, physicians, and others create values easily and behave differently in offline and online settings [2,19,20]. Therefore, combining the above 2 categories, 4 dimensions of VCCH are proposed according to whether VCCH happens inside or outside the hospital and offline or online. The dimensions are summarized in Multimedia Appendix 5.

### Inside-Hospital Offline Value Cocreation in Health Care

In this dimension, value is cocreated by participants within the hospital and does not involve any health information technology. The main participants include patients, doctors, nurses, and other hospital staff. Several behaviors are discussed in this dimension, including value cocreation behavior, patient participation behavior, patient responsible behavior, and customer effective behavior.

For value cocreation behavior, Yi and Gong [21] identified 2 types: participation behavior and citizenship behavior, while Olsson [22] used complaints and feedback to measure value cocreation behaviors. Regarding patient participation behavior, it is reflected as information sharing [23-26], information seeking [23,26], coproducing, cooperating [6], maintaining interpersonal interaction [6,24,26,27], and enjoying spending time with other patients at the hospital [24], etc. Patient responsible behavior includes following the doctor’s instructions to take prescribed medication and complying with the doctor’s diagnosis-related recommendations [23,25]. Customer effective behavior includes patients’ scientific use of health-related instruments and tracking of disease-related indicators according to the guidance of medical professionals [28].

### Inside-Hospital Online Value Cocreation in Health Care

With the application of information technology in health care, health information systems (HIS) including electronic health records are widely used in hospitals to create patient value by health care participants. HIS enables patients to increase their knowledge and connect with their physicians, while HIS makes it easy for health professionals to access information and facilitate communication with patients [29]. Therefore, HIS helps build a network of interdependent health care participants to cocreate their value by facilitating interactions between participants [30].

According to Beirão et al [11], with the support of HIS, VCCH could be divided into 3 levels from the ecosystem perspective: macro, meso, and micro. At the macro level, the main participants are governmental departments, ministries of health, and other organizations that shape national health policies. At this level, the context is the ecosystem within which VCCH happens constantly. At the meso level, the main parties are hospitals, primary care units, and health organizations. At this level, parties serve one another directly and indirectly to cocreate patient value. At the micro level, the main actors are health professionals, patients, and their families. At this level, VCCH generally occurs between actors’ dyads.

### Outside-Hospital Offline Value Cocreation in Health Care

In this dimension, VCCH happens outside the hospital and does not involve information technologies such as the internet. The main participants in this dimension are patients, families, and their friends. Based on the literature review, VCCH is reflected as patient participation behavior, health-related complementary behavior, and patient self-administration in this dimension.

Patient participation behavior could be indicated as sharing worries and anxieties with others, maintaining good relationships with family and friends [24], and seeking and sharing health information with others [6,31]. Health-related complementary behavior includes monitoring and maintaining a healthy diet, exercising, changing the ways of doing things and interacting with others, and dancing and spending time with children to reduce anxiety [31]. Similar concepts like health behavior changes and complementary therapies [6] have been proposed in previous literature. Health behavior changes contain physical and dietary health behavior changes, while complementary therapies refer to improving one’s health status by taking complementary medicines, exercise, yoga, meditation, and other activities [6]. To assess whether innovative therapeutic solutions create value for patients and health systems, Spano et al [32] used patients’ self-administration to represent VCCH. Their self-administration is to administer a patient’s therapy, such as subcutaneous injection, at home.

### Outside-Hospital Online Value Cocreation in Health Care

In this dimension, information technology has been applied outside hospital for health purposes. Many digital apps such as digital health platforms have become popular for patients to seek and use health information. Digital health platforms help patients connect with others, share information and experiences, and receive support for good health outcomes.

According to Presti et al [33], value cocreation online can be represented as customer engagement, which includes affection, activation, and cognitive dimensions. The affection dimension is the extent to which the patient influences the platform or health care services during the platform interaction; the activation dimension is defined as time, energy, and the energy spent by the patient during the interaction; and the cognitive dimension refers to the cognitive processing activated by the patient during the platform interaction.

From the perspective of the service provider and receiver, 3 types of engagement practices are proposed: information, advising, and empathy practices [34]. In addition, digital information search [35], collation of health information [6,31], and knowledge contribution [36] to other members of the online platform also are part of patient engagement.

Beside customer engagement, connecting with others could also convey VCCH in this dimension [6]. To be specific, patient connection with others could be shown as their experience using online platform [37] and maintaining ongoing relationships among online community members [36].

### Antecedents of Value Cocreation in Health Care

To review the antecedents of VCCH, we categorize the previous literature according to 2 dimensions: actors and settings. For actors, as mentioned in the previous section, several actors, including patients, health care professionals, families, and friends, are involved in value cocreation. However, previous literature studies the antecedents mainly from the perspectives of 2 actors: patient and health care professional. For settings, the widespread use of information technologies, especially internet technology in health care, plays an important role in the cocreation of patient value. Therefore, we could classify the antecedents into 4 types: patient offline perspective, patient online perspective, health care professional offline perspective, and health care professional online perspective. The antecedents are all summarized in Multimedia Appendix 6.

### Patient Offline Perspective

In this perspective, factors that influence VCCH are studied from the patient perspective in the offline setting. We divide the factors into 2 types according to their attributes: intrinsic factors and extrinsic factors. Intrinsic factors are mainly about patients themselves. For example, positive intrinsic motivation [28,38] and positive emotions [9] are found to facilitate patient participation. Patient empowerment is shown to promote value cocreation behaviors. Patients’ trust in physicians [26,39], provider-patient orientation [40], and patient pre-encounter actor value needs [39] affect the cocreation of patient value, which in turn affects the service experience and satisfaction. Finally, personality traits (including agreeableness and extraversion), personal values [10], and gender [22] of patients also have effects on value cocreation behaviors.

Extrinsic factors are factors outside patients. For example, the interaction between patients and physicians, other patients, and their families or friends positively affects value cocreation behaviors [23,24,31]. Physician performance is found to influence patient information seeking, information sharing, and responsible behavior. Patient perceived transparency is shown to effectively reduce information asymmetry between doctors and patients and influences patients’ perceived value and satisfaction [41]. Finally, negative factors including lack of empathy, support, and courtesy from physicians and stereotyping of health professionals can lead to patient dissatisfaction and even complaint behaviors [22,40].

### Patient Online Perspective

In this perspective, factors are studied about patients in online settings such as blogs, patient forums, online health communities, and internet hospitals. To be specific, social support, which includes information support, emotional support, and instrumental support, is found to facilitate users’ social, functional, and affective values through interaction or cocreation activities among users in online health communities [37,42]. Social identity, which is predicted by integrity trust, benevolence trust, shared vision, and shared language, is found to drive patients’ value cocreation activities such as knowledge contribution and continuous willingness to participate [36]. Finally, different types of information processing have also been shown to affect the generation of different kinds of value in online health communities [43]. Meanwhile, users’ community experiences and social exclusion have often been used as moderating variables.

### Health Care Professional Offline Perspective

From this perspective, factors are studied for health care professionals in offline settings. The main health care professionals involved are doctors and nurses. For doctors, factors determining health care professional behaviors and contributing to value creation include information being provided, patient engagement, trust, physical environment, and collaboration [26]. In addition, a patient’s trust in the physician facilitates the physician’s management of the patient’s condition which facilitates VCCH. For nurses, patient participation, length of stay, and first inpatient stay are found to influence nurses’ value creation behaviors such as work engagement, job satisfaction, and helping behaviors [44].

### Health Care Professional Online Perspective

In this perspective, factors are studied for health care professionals in online settings. Health information technologies in the health care systems in the world are implemented to address health care challenges, including aging populations living with long-term conditions, persistent variations in the quality of care, and health care cost rising. Health care professionals use of health information technologies in their work can be compulsory or voluntary. The use of health information technologies like electronic health records has becoming a key factor that promotes VCCH by supporting and facilitating multiple interactions among participants at different levels, enabling resource access, sharing, reorganization, and even resource monitoring and institutional generation [11]. Whether a physician is successful in obtaining electronic health information about a patient and the degree of mastery, portability, and credibility of advanced technology will affect the physician’s grasp of patient disease and thus the value cocreation [45].

### Consequences of Value Cocreation in Health Care

In VCCH, patients and health care professionals are the main participants, and the cocreated value or other consequences would be mainly distributed between them. Therefore, we could divide the consequences of VCCH into 2 types: patient value and health care professional value. The consequences are summarized in Multimedia Appendix 7.

### Patient Value

Patient value is the valuable consequence of value cocreation for patients. Previous literature has discussed many consequences related to health outcomes, service, or overall value. On the health outcomes related consequences, health conditions [26,28,35] and well-being [31,35,40,46] are discussed and found to be helpful in determining the effectiveness of clinical interventions and quality. These consequences can be reflected in patients feeling more energetic, feeling better, and having blood pressure return to normal levels, etc. Moreover, patient compliance, which could predict health outcomes, is also in focus [27]. Finally, health expenditures could reflect patient value according to previous literature.

For service-related consequences, service experience is emphasized as the final goal of value creation [11]. Meanwhile, perceived service quality [9,24,41] and patient satisfaction [24,39-41,47,48], positive word-of-mouth, customer loyalty [10,24], and service engagement [26,27] are also considered as important service-related consequences of VCCH.

Finally, some studies investigate patient value directly. For example, cure and care values are shown to be created by patients through posting and communications with others in online health communities [43]. Perceived value, which includes perceived benefits and perceived cost, could be the result of a patient experience situation in the service contact [37] or patient participation in services [38,49]. Perceived value is also divided into process value and outcome value [23].

### Health Care Professional Value

The main health professionals studied are doctors and nurses. For the value of doctors, effectiveness and efficiency of their work can be achieved by effective value cocreation activities [11]. Work effectiveness includes higher diagnostic accuracy, prescription accuracy and safety, prescription compliance, and higher patient orientation while work efficiency means fewer repetitive tests, shorter consultation times, faster work procedures, and avoidance of lost documents. For nurses, the value they created could be predicted by patients’ experience of their hospital admission and length of stay, reflected by job satisfaction and technical skills among the nurses [44].

### Integrative Framework for Value Cocreation in Health Care

**Figure 4:**
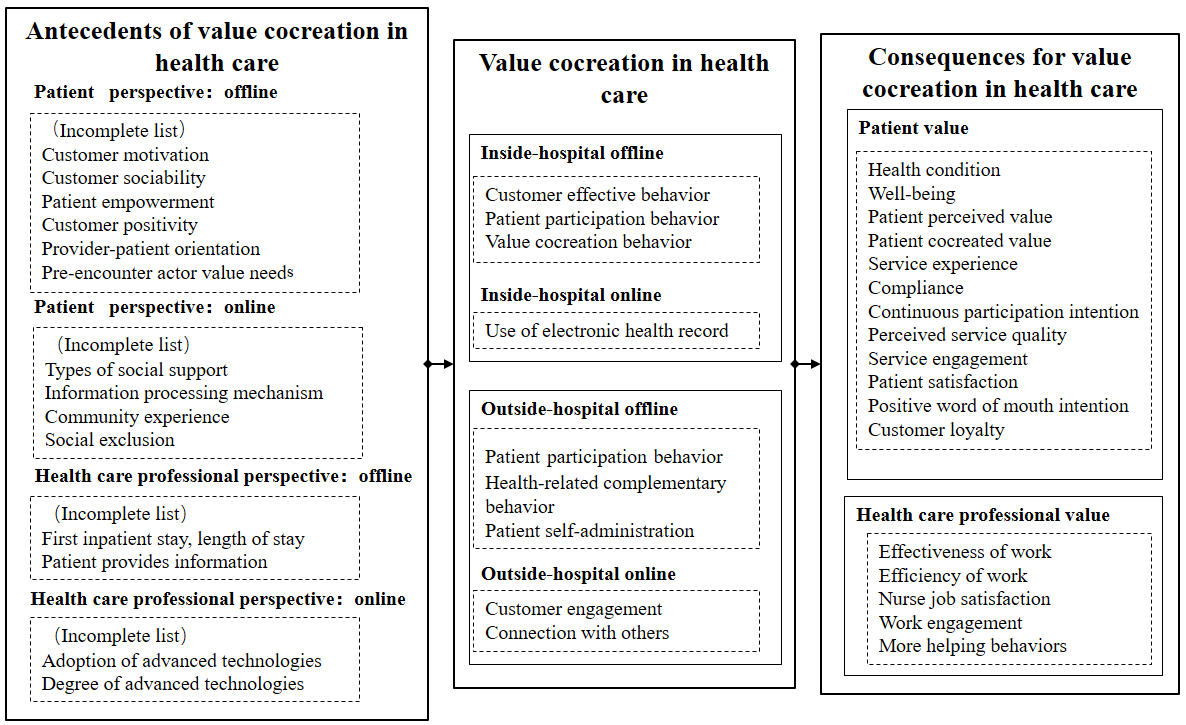
Framework of value cocreation in health care based on social cognitive theory.

To provide a holistic view of VCCH, we propose an integrative framework by summarizing all of the reviews based on social cognitive theory (Figure 4). The proposed integrative framework could have sufficient impact on journal publication in several ways.

First, the factors and their relationships related to VCCH are systematically mapped and visualized in the framework. In the integrative framework of VCCH, the relationships among antecedents, consequences, and dimensions of VCCH are revealed. This framework could then be used to explain, predict, and evaluate VCCH systematically and comprehensively. Meanwhile, the framework of VCCH also makes the formation process of values for patients and health care professionals through their cocreation transparent. Therefore, this framework helps open the black box of VCCH.

Second, the framework provides a novel theoretical perspective for VCCH. In previous literature, SDL serves as the dominant theoretical perspective for VCCH. However, this framework is proposed based on social cognitive theory, which could bring a novel theoretical perspective for VCCH. Social cognitive theory proposes that people’s behaviors results from learning in social context, while environmental and personal factors would determine the whole learning process. Therefore, VCCH behaviors could be treated as learned behaviors and would be determined by environmental and personal factors.

Finally, the framework implies many future research directions. Based on the research gaps and relationships conveyed by the framework, many future research directions exist, such as alternative research methods and applications in different contexts. VCCH in online settings or mixed settings should be further investigated, and other types of values creation should be considered.

## Discussion

### Principal Findings

The purpose of this study was to conduct a systematic review of the literature on VCCH. In this review, we focused on empirical studies about VCCH. After a comprehensive search of the databases, 28 journal articles were identified by rigorous application of inclusion and exclusion criteria.

We present our review results from 6 aspects: publication years and journals, research methods, theories, dimensions of VCCH, antecedents of VCCH, and consequences of VCCH. To be specific, we depict the research methods and theories used in VCCH literature. Meanwhile, we categorize the VCCH, its antecedents, and consequences into several dimensions according to 2 main criteria: actors and settings. Actors are mainly patients and health care professionals, while settings include hospitals and the internet. Finally, to summarize our literature review systematically and provide theoretical insights, we propose an integrative framework to map the literature grounded on social cognitive theory.

### Implications

We believe that the findings of this study could convey several implications for both theories and practice. For theoretical implications, this study provides a comprehensive and systematic literature review of VCCH. With the application of SDL in health care, studies about value cocreation are emerging. However, none of the previous literature has provided a systematic summary to reflect the trends, gaps, and directions of VCCH research from different perspectives. Thus, we conducted a systematic review of literature on VCCH. Based on our review, we presented the trends of publication, research methods, and theories of VCCH. Meanwhile, the gaps and research directions of VCCH could be analyzed and speculated about according to our proposed dimensions, antecedents, and consequences of VCCH. We not only summarize previous literature but also propose an integrative framework of VCCH to map previous literature and guide future research. We integrate the dimensions, antecedents, and consequences of VCCH into the integrative framework to reveal the relationships among the 3 aspects of VCCH from a social cognitive theory perspective.

For practical implications, our systematic review confirms the effectiveness of VCCH. Our study provides a higher quality of evidence compared with single studies about VCCH. Policy makers and health care practitioners can encourage patients and health care professionals to create values together. The uncovered dimensions of VCCH could be the objectives of value cocreation activities. Our summarized dimensions provide a systematic map of VCCH to guide value cocreation practices and improve the feasibility of policies about VCCH. Finally, the antecedents could be the predictors of VCCH. Thus, policy makers and health care practitioners could take action according to antecedents to promote VCCH.

### Future Research Opportunities

Our systematic literature review enables us to highlight some opportunities for future research. First, other research methods could be used to conduct more rigorous studies of VCCH. Different methods have advantages and disadvantages in investigating VCCH. Although questionnaires and surveys have dominated previous literature, other methods including randomized controlled trial (RCT) or experiment, case study, or secondary data analysis may have more advantages [50]. For example, compared with surveys, experiments and RCTs have advantages in internal validity that provide strong evidence of causality by minimizing various possible biases, balancing out confounding factors, and improving the validity of statistical tests. Meanwhile, a case study could provide comprehensive and rich information of research objects and inspire unique insights. Finally, secondary data analysis allows investigation of the dynamics of research objects. Therefore, other research methods could be used to better understand VCCH. Because of the complexity of related questions, methods could be used simultaneously rather than by themselves [51].

Second, contextualized and special developed theories are needed for VCCH. Theories could help solve research questions by providing support for analysis, explaining and predicting the research questions [52]. Contextualized and special developed theories could better solve questions related to VCCH compared with theories borrowed from other contexts or domains since contextualized or special developed theories could better reflect the context and regulation of VCCH [53]. The reflection of context of VCCH could capture the person-situation interaction, linkage between personal activities and value creation, and variation among different constructs and convey the application feasibility of study of VCCH [54]. In VCCH, although SDL is found to be the main theoretical foundation in previous literature, contextualization of SDL or building special theories promotes the understanding of VCCH.

Third, VCCH in online settings or mixed settings should be further investigated. With the application of emerging information technologies in health care including artificial intelligence, big data, and cloud computing, creation of patient value has been changed [55]. Many health care activities happen not only in offline settings but also in online settings or other settings like telemedicine, eHealth, or mobile health in recent years. For example, patients and physicians interact with each other in online health communities to create value by solving the health concerns of patients and rewarding physicians with money or reputation [56]. Meanwhile, electronic health records facilitate the cocreation of value by allowing patients to record their health information and physicians to know patients’ information comprehensively. Finally, telemedicine help create value by letting patients and physicians contact each other remotely. Characteristics of different settings would bring different opportunities and challenges to health care and change value cocreation. Therefore, it will be necessary to investigate VCCH in online or mixed settings.

Fourth, more types of values should be considered. Based on our review, values for patients, doctors, and nurses are explored in previous literature. However, more actors have been participating in value cocreation, like friends, families, peers, and allied health professionals, and what they value should be different from patients and doctors; few studies in the previous literature concern their values in the cocreation process. In addition, activities and interactions between doctors and patients could translate into value of the health care organization or even the whole health care system. Values and value cocreation for health professionals and patients in primary, secondary, and tertiary levels of care could be different and are worthy of investigation in the future. Therefore, no matter the value of the patient, health care professional, or individual, value at the organizational or health care system level should also be regarded to better reflect the effectiveness of VCCH.

Finally, since we map previous literature onto social cognitive theory, many related factors could be considered in future study. According to social cognitive theory, factors could be divided into 5 categories: outcome expectancies, social learning, self-efficacy, self-regulation, moral engagement, and other environmental factors [57]. All factors are important antecedents of people’s value cocreation behaviors. All the factors are important antecedents of people’s value cocreation behaviors. Outcome expectancy is defined as an expectation that an outcome will follow a given behavior [58], while social learning is learning from the knowledge and experience of others we know and trust [59]. Self-efficacy is defined as beliefs about one’s ability to perform a specific behavior [60], while self-regulation refers to self-generated thoughts, affects, and behaviors that are systematically oriented toward attainment of one’s goals [61]. On environmental factors, they could contain social networks, moral engagement, and culture, etc. Therefore, we would check previously studied factors and see whether any factors from these 5 categories are unexplored and feasible to be studied.

### Limitations

There are limitations of this systematic review. Our results are based on journal articles that fit our inclusion criteria and do not include other types of literature (eg, conference proceedings), which may miss some recent research. Thus, the search scope of the literature could be further expanded. Another limitation is that this study primarily screened for studies strictly related to VCCH. Other types of studies like conceptual research or papers that focus on other actors than patients in VCCH may also provide some insights.

### Conclusion

This study presents findings and implications of a systematic review of 28 journal articles for value cocreation in health care. Our review findings are presented as publication years and journals, research methods, theories, dimensions of VCCH, antecedents of VCCH, and consequences of VCCH. Based on review findings, we proposed an integrative framework based on social cognitive theory to systematically map the literature and reveal the whole value cocreation process in health care. We believe that our literature review and theoretical framework could contribute to the deep understanding of VCCH and inspire more research in the future.

## Appendix

Multimedia Appendix 1 Preferred Reporting Items for Systematic Reviews and Meta-Analyses checklist.

Multimedia Appendix 2 Quality assessment of included studies.

Multimedia Appendix 3 List of published journals.

Multimedia Appendix 4 Theoretical foundations in the literature.

Multimedia Appendix 5 Dimensions of value cocreation in health care.

Multimedia Appendix 6 Antecedents of value cocreation in health care.

Multimedia Appendix 7 Consequences of value cocreation in health care.

## Abbreviations

HIS: health information systems
MMAT: Mixed Methods Appraisal Tool
PRISMA: Preferred Reporting Items for Systematic Reviews and Meta-Analyses
RCT: randomized controlled trial
SDL: service-dominant logic
VCCH: value cocreation in health care
