# Supplementary material for: Value Cocreation in Health Care: Systematic Review
Source: J Med Internet Res. 2022 Mar 25;24(3):e33061. doi: 10.2196/33061 (PMC8994154; doi:10.2196/33061)
Supplement: Multimedia Appendix 2 [file jmir_v24i3e33061_app2.docx]

**Multimedia Appendix 2.** Quality assessment of included studies.

| Type of Study | Study | Screening Questions | | Methodological Quality Criteria | | | |
| --- | --- | --- | --- | --- | --- | --- | --- |
| 1. Qualitative |  | Are there clear qualitative and quantitative research questions(or objectives) or a clear mixed methods question(or objective)？ | Do the collected data allow address the research question(objective)？ | 1.1. Are the sources of qualitative data (archives, documents, informants, observations) relevant to address the research question (objective)？ | 1.2. Is the process for analyzing qualitative data relevant to address the research question (objective)？ | 1.3. ls appropriate consideration given to how findings relate to the context, e.g.， the setting, in which the data were collected？ | 1.4. Is appropriate consideration given to how findings relate to researchers’ influence, e.g.， through their interactions with participants？ |
|  | Janet R. McColl-Kennedy, et al. (2012) | Yes | Yes | Yes | Yes | Yes | Yes |
|  | Susan Stewart Loane, et al. (2015) | Yes | Yes | Yes | Yes | Yes | Yes |
|  | Erik Masao Olsson (2016) | Yes | Yes | Yes | Yes | Yes | Yes |
|  | Kofi Osei-Frimpong, et al.(2017) | Yes | Yes | Yes | Yes | Unclear | Yes |
|  | Beirao, Gabriela, et al.(2017) | Yes | Yes | Yes | Yes | Yes | Yes |
|  | Spano, Rosanna, et al.(2018) | Yes | Yes | Yes | Yes | Yes | Yes |
|  | Stadtelmann, Michael, et al.(2019) | Yes | Yes | Yes | Yes | Yes | Yes |
| 5.  Mixed methods | Janet R. McColl-Kennedy, et al. (2017) | Yes | Yes | Yes | Yes | Unclear | Yes |
| 5.  Mixed methods | Osei-Frimpong, Kofi, et al.(2018) | Yes | Yes | Yes | Yes | Yes | Yes |
| 5.  Mixed methods | Zhao, Jing, et al. (2015) | Yes | Yes | Yes | Yes | Yes | Yes |
| 4. Quantitative descriptive |  | Are there clear qualitative and quantitative research questions(or objectives) or a clear mixed methods question(or objective)？ | Do the collected data allow address the research question(objective)？ | 4.1. Is the sampling strategy relevant to address the quantitative research | 4.2. Is the sample representative of the population understudy？ | 4.3. Are measurements appropriate (clear origin, or validity known or standard instrument )？ | 4.4. Is there an acceptable response rate (60% or above )？ |
|  | Le Nguyen Hau (2016) | Yes | Yes | Yes | Yes | Yes | Yes |
|  | Gallan, Andrew S (2013) | Yes | Yes | Yes | Unclear | Yes | No |
|  | Ferguson, Ronald J (2010) | Yes | Yes | Yes | No | Yes | Yes |
|  | Dahl, Andrew J (2018) | Yes | Yes | Yes | Yes | Yes | No |
|  | Uzay Damali , et al. (2016) | Yes | Yes | Yes | Yes | Yes | No |
|  | Bin Ding, et al. (2019) | Yes | Yes | Yes | Yes | Yes | Yes |
|  | DonHee Lee (2019) | Yes | Yes | Yes | Yes | Yes | No |
|  | Russo, Giuseppe (2019) | Yes | Yes | Yes | Yes | Yes | Yes |
|  | Letizia Lo Presti (2019) | Yes | Yes | Yes | Yes | Yes | Yes |
|  | Kofi Osei-Frimpong (2016) | Yes | Yes | Yes | Yes | Yes | Yes |
|  | Yang, Yongheng (2018) | Yes | Yes | Yes | Yes | Yes | Yes |
|  | Kofi Osei-Frimpong (2017) | Yes | Yes | Yes | Yes | Yes | Yes |
|  | Jiyoung Kim (2018) | Yes | Yes | Yes | Yes | Yes | Yes |
|  | Le Nguyen Hau, et al. (2017) | Yes | Yes | Yes | Yes | Yes | yes |
|  | Huetten, Antje Sarah Julia, et al. (2019) | Yes | Yes | Yes | Yes | Yes | No |
|  | Van Oerle, Sarah, et al.(2018) | Yes | Yes | Yes | yes | Yes | not applicable |
|  | Liu, Wenlong, et al. (2019) | Yes | Yes | Yes | yes | Yes | yes |
|  | Jiyoung Kim (2019) | Yes | Yes | Yes | Yes | Yes | Yes |
| 5.  Mixed methods | Janet R. McColl-Kennedy, et al. (2017) | Yes | Yes | Yes | Yes | Yes | Yes |
| 5.  Mixed methods | Osei-Frimpong, Kofi, et al.(2018) | Yes | Yes | Yes | Yes | Yes | Yes |
| 5.  Mixed methods | Zhao, Jing, et al. (2015) | Yes | Yes | Yes | Yes | Yes | Unclear |
| 5.  Mixed methods |  | Are there clear qualitative and quantitative research questions(or objectives) or a clear mixed methods question(or objective)？ | Do the collected data allow address the research question(objective)？ | 5.1. Is the mixed methods research design relevant to address the qualitative and quantitative research questions (or objectives)， or the qualitative and quantitative aspects of the mixed methods question (or objective)？ | 5.2. Is the integration of qualitative and quantitative data（or results ）relevant to address the research question (objective)？ | 5.3. Is appropriate consideration given to the limitations associated with this integration, e.g., the divergence of qualitative and quantitative data (or results) in a triangulation design？ |  |
|  | Janet R. McColl-Kennedy, et al. (2017) | Yes | Yes | Yes | Yes | No |  |
|  | Osei-Frimpong, Kofi, et al.(2018) | Yes | Yes | Yes | Yes | No |  |
|  | Zhao, Jing, et al. (2015) | Yes | Yes | Yes | Yes | No |  |
