# Supplementary material for: Value Cocreation in Health Care: Systematic Review
Source: J Med Internet Res. 2022 Mar 25;24(3):e33061. doi: 10.2196/33061 (PMC8994154; doi:10.2196/33061)
Supplement: Multimedia Appendix 3 [file jmir_v24i3e33061_app3.docx]

**Multimedia Appendix 3.** List of published journals.

| Journal Name | Studies |
| --- | --- |
| Journal of Service Management | [10, 11, 36, 40] |
| Journal of Service Theory and Practice | [24, 38, 41] |
| Service Business | [23, 25, 45] |
| Sustainability | [33, 46, 48] |
| International Journal of Environment Research and Public Health | [42, 44] |
| International Journal of Pharmaceutical and Healthcare Marketing | [34, 39] |
| BMC Health Services Research | [32] |
| Journal of Operations Management | [28] |
| Journal of Business Research | [31] |
| Journal of the Academy of Marketing Science | [9] |
| The Journal of Consumer Affairs | [35] |
| Journal of Service Research | [6] |
| Journal of Macromarketing | [37] |
| International Journal of Health Care Quality Assurance | [22] |
| Technological Forecasting & Social Change | [27] |
| Journal of Nonprofit & Public Sector Marketing | [26] |
| Psychology & Marketing | [43] |
