# Supplementary material for: Value Cocreation in Health Care: Systematic Review
Source: J Med Internet Res. 2022 Mar 25;24(3):e33061. doi: 10.2196/33061 (PMC8994154; doi:10.2196/33061)
Supplement: Multimedia Appendix 4 [file jmir_v24i3e33061_app4.docx]

**Multimedia Appendix 4.** Theoretical foundations in the literature.

| Theory | Studies |
| --- | --- |
| Service-Dominant Logic (SDL) | [6, 28, 34, 35, 38, 41, 44] |
| Practice Theory | [6, 31] |
| Social Identity Theory（SIT） | [36] |
| Construal Level Theory（CLT) | [31] |
| Self-Regulation Theory (SRT) | [31] |
| Consumer Culture Theory (CCT) | [6] |
| Self-Awareness Theory | [43] |
| Social Cognitive Theory | [24] |
| Organizational Support Theory | [42] |
| Self-Determination Theory (SDT) | [38] |
| Broaden-and-Build Theory of Positive Emotions | [9] |
| Customer Training and Education (CTE) | [28] |
