# Supplementary material for: Value Cocreation in Health Care: Systematic Review
Source: J Med Internet Res. 2022 Mar 25;24(3):e33061. doi: 10.2196/33061 (PMC8994154; doi:10.2196/33061)
Supplement: Multimedia Appendix 5 [file jmir_v24i3e33061_app5.docx]

**Multimedia Appendix 5.** Dimensions of value cocreation in health care.

| **Dimensions** | **Reflections** | **Indicators** | **Actors** |
| --- | --- | --- | --- |
| Inside-  Hospital Offline | Customer effective behavior [28] | —— | Doctors, nurses and other staff in hospital |
|  | Patient participation behavior  [9, 23, 25, 26, 38, 40, 44-46] | - Information sharing [23-26] |  |
|  |  | - Shared-decision making [24, 27, 38] |  |
|  |  | - Coproduce [6, 31] |  |
|  |  | - Collaboration [26] |  |
|  |  | - Cooperating [6, 26] |  |
|  |  | - Co-learn [6, 31] |  |
|  |  | - Interactions with doctors [6, 24, 26, 27] |  |
|  |  | - Information seeking [23] |  |
|  |  | - Enjoyed spending time with other patients at the hospital[24] |  |
|  |  | - Voluntary in-role feedback [25] |  |
|  |  | - Responsible behavior [23, 25] |  |
|  |  | - Intention to participate in value co-creation [45] |  |
|  |  | - patient efforts in co-production of services [9, 40, 44, 46] |  |
|  | Value co-creation behavior | - Patient participation behavior & Patient citizenship behavior [48] |  |
|  |  | - Complaints and feedback [22] |  |
| Inside-  Hospital Online | Electronic Health Record use | - Resource access, resource sharing, and resource recombination [11] | The users of hospital information systems |
| Outside-hospital Offline | Patient participation behavior | - Co-learn [6, 31] | Patients, families, friends, caregivers, and health professionals in supportive entities |
|  |  | - Collate [6, 31] |  |
|  |  | - Sharing worries and anxieties with others [24] |  |
|  |  | - Maintain good relationship with others [24] |  |
|  | Health-related complementary behaviors | - Monitoring and maintaining a healthy diet and exercise [31] |  |
|  |  | - Change ways of doing things [6, 31] |  |
|  |  | - Distracting activities [31] |  |
|  |  | - Combining complementary therapies [6] |  |
|  |  | - Health behavior changes [35] |  |
|  | Patient self-administration [32] | —— |  |
| Outside-hospital Online | Customer engagement [33] | - Digital information search [35] | Users of health internet applications |
|  |  | - Collate [6, 31] |  |
|  |  | - Information-, advising- and empathy-practices [34] |  |
|  |  | - Customer interactions [42] |  |
|  | Connecting with others | - Membership continuance intention [36] |  |
|  |  | - Customer experience [37] |  |
