# Supplementary material for: Value Cocreation in Health Care: Systematic Review
Source: J Med Internet Res. 2022 Mar 25;24(3):e33061. doi: 10.2196/33061 (PMC8994154; doi:10.2196/33061)
Supplement: Multimedia Appendix 6 [file jmir_v24i3e33061_app6.docx]

**Multimedia Appendix 6.** Antecedents of value cocreation in health care.

| **Antecedents** | | | |
| --- | --- | --- | --- |
|  | | **Setting dimension** | |
|  |  | **Offline** | **Online** |
| **Actor dimension** | Patient perspective | - Customer motivation [28, 38] | - Social identity [36] |
|  |  | - Customer sociability [10] | - Types of social support [37, 42] |
|  |  | - Patient empowerment [48] | - Information processing mechanism [43] |
|  |  | - Customer positivity [9] | - Community experience [43] |
|  |  | - Provider-patient orientation [40] | - Social exclusion [42] |
|  |  | - Pre-encounter actor value needs [39] |  |
|  |  | - Level of trust [26, 39] |  |
|  |  | - Gender [22] |  |
|  |  | - Role clarity [26, 28] |  |
|  |  | - Provide enabling environment [26] |  |
|  |  | - Care delivery approach [26, 39] |  |
|  |  | - Transparency [41] |  |
|  |  | - Frontline interaction [23] |  |
|  |  | - Object of interactions [24, 31] |  |
|  |  | - Primary care provider access [35] |  |
|  |  | - Physician resource offering [46] |  |
|  |  | - Stereotypes toward health care professionals [40] |  |
|  |  | - Lack of information, lack of consideration, lack of empathy, lack of support and courtesy [22] |  |
|  | Health care professional perspective | - First inpatient stay, length of stay [44] | - Adoption of advanced technologies [11] |
|  |  | - Patient provides information, level of patient engagement, level of trust, provide the enabling environment, maximum collaboration [26] | - Degree of advanced technologies, degree of easy access, degree of credibility [45] |
