# Supplementary material for: Value Cocreation in Health Care: Systematic Review
Source: J Med Internet Res. 2022 Mar 25;24(3):e33061. doi: 10.2196/33061 (PMC8994154; doi:10.2196/33061)
Supplement: Multimedia Appendix 7 [file jmir_v24i3e33061_app7.docx]

**Multimedia Appendix 7.** Consequences of value cocreation in health care.

| **Consequences** | |
| --- | --- |
| **patient value** | **healthcare professional value** |
| - Health conditions [26, 28, 35] | - Effectiveness of work [11] |
| - Well-being [31, 35, 40, 46] | - Efficiency of work [11] |
| - Patient perceived value [23, 38, 41] | - Nurse job satisfaction [44] |
| - Patient co-created value[39, 43, 46] | - Work engagement [44] |
| - Service experience[10, 11, 26] | - More helping behaviors [44] |
| - Compliance [27] |  |
| - Continuous participation intention[42] |  |
| - Perceived service quality [9, 24, 41] |  |
| - Service engagement [26, 27] |  |
| - Health expenses[26, 28] |  |
| - Patient satisfaction [24, 39-41, 47, 48] |  |
| - Positive WOM intention/customer loyalty [10, 24] |  |
